# Catheter Ablation of Arrhythmias Editors: Douglas P. Zipes, MD, Michel Haissaguerre, MD. 2nd Edition, 2002 Futura Publishing Company, Inc

**Published:** 2002-01-01

**Authors:** Kamal K Sethi

**Affiliations:** Director, Interventional Cardiology and Arrhythmia Service, Sir Ganga Ram Hospital, New Delhi, India

Since the publication of the first edition of this book eight years ago, considerable new information has been acquired about mechanisms of arrhythmias, pathways and sites for ablation, and techniques of ablation. Publication of the second edition is, therefore, timely and contemporary. With chapters contributed by well recognized international experts in arrhythmia management, the book is a practical and useful reference for physicians engaged in catheter ablation. It brings together presentations made at Bordeaux Symposium held in May 2001.

Of particular interest to the basic scientist are mechanisms of reentrant arrhythmia, especially ventricular tachycardia, studied in experimental, perfused preparations. Chapters on minimal electrophysiological assessment and mapping techniques before ablation are brief but informative. Curative treatment of atrial fibrillation, a challenge for the last decade, has been described succinctly, focussing both on pulmonary venous as well as linear ablation techniques. Adequate emphasis has also been laid on typical and atypical atrial flutter and focal atrial tachycardias, written individually in separate chapters.

The classic arrhythmias for which catheter ablation was initiated, viz, atrioventricular nodal reentry, accessory pathways and Mahaim fibres are also well represented. The approach to ventricular tachycardia related to structural heart disease, with its attendant complexities, and advances in newer mapping techniques, is comprehensively described. Finally, as the emotional impact of our ability to abolish conduction over accessory pathways still holds, ventricular fibrillation is the new, emerging challenge.

The book has clear and concise figures, including coloured examples of relevant imaging techniques. The style is very readable; the information can be absorbed easily. It is certainly an important addition to the current knowledge about the source and treatment of cardiac arrhythmias.

